# Phenylglyoxal-Based Visualization of Citrullinated Proteins on Western Blots

**DOI:** 10.3390/molecules20046592

**Published:** 2015-04-14

**Authors:** Sanne M. M. Hensen, Wilbert C. Boelens, Kimberly M. Bonger, Remco T. P. van Cruchten, Floris L. van Delft, Ger J. M. Pruijn

**Affiliations:** 1 Department of Biomolecular Chemistry, Institute for Molecules and Materials, Radboud Institute for Molecular Life Sciences and Netherlands Proteomics Centre, Radboud University Nijmegen, P.O. Box 9101, NL-6500 HB Nijmegen, The Netherlands; E-Mails: sannehensen@gmail.com (S.M.M.H.); w.boelens@ncmls.ru.nl (W.C.B.); k.bonger@science.ru.nl (K.M.B.); remcovancruchten@gmail.com (R.T.P.C.); 2 Department of Synthetic Organic Chemistry, Institute for Molecules and Materials, Radboud University Nijmegen, P.O. Box 9101, NL-6500 HB Nijmegen, The Netherlands; E-Mail: f.vandelft@synaffix.com

**Keywords:** citrulline, peptidylarginine deiminases, anti-modified citrulline antibodies, phenylglyoxal, azide-alkyne cycloaddition, rheumatoid arthritis

## Abstract

Citrullination is the conversion of peptidylarginine to peptidylcitrulline, which is catalyzed by peptidylarginine deiminases. This conversion is involved in different physiological processes and is associated with several diseases, including cancer and rheumatoid arthritis. A common method to detect citrullinated proteins relies on anti-modified citrulline antibodies directed to a specific chemical modification of the citrulline side chain. Here, we describe a versatile, antibody-independent method for the detection of citrullinated proteins on a membrane, based on the selective reaction of phenylglyoxal with the ureido group of citrulline under highly acidic conditions. The method makes use of 4-azidophenylglyoxal, which, after reaction with citrullinated proteins, can be visualized with alkyne-conjugated probes. The sensitivity of this procedure, using an alkyne-biotin probe, appeared to be comparable to the antibody-based detection method and independent of the sequence surrounding the citrulline.

## 1. Introduction

Peptidylarginine deiminases (PADs) catalyze the conversion of the guanidine group of arginine residues present in polypeptides to a ureido group, a process known as citrullination [1]. This chemical alteration leads to a loss of charge and may affect the structure and function of polypeptides. Carbamylation, the conversion of peptidyllysine into peptidylhomocitrulline, also results in the generation of a ureido group; the side chain of citrulline and homocitrulline differ by just one methylene moiety. To study the biological role of citrullination in both health and disease, a series of methods has been developed to detect this post-translational modification [2]. An often used method for detection of (homo)citrullinated proteins by western blot analysis is based on the specific chemical modification of the (homo)citrullines with diacetyl monoxime (DAMO) and antipyrine in an acidic environment, followed by visualization of the modified (homo)citrullines by anti-modified citrulline (AMC) antibodies, originally described by Senshu and coworkers [3]. However, the production of new AMC antibodies proved to be difficult and for this reason there is a need for antibody-independent strategies to detect citrullinated proteins. 

Phenylglyoxal (PG) is chemically related to DAMO and reacts specifically with citrulline and most likely also homocitrulline residues under highly acidic conditions. Tutturen and colleagues [4] applied this reaction to enrich citrullinated peptides for mass spectrometry analysis using beads that were functionalized with 4-hydroxy-PG via a cleavable linker. Recently, a PG-rhodamine probe has been developed to allow visualization of citrullinated proteins after separation by sodium dodecyl sulfate polyacrylamide gel electrophoresis (SDS-PAGE) [5]. A drawback of this direct citrulline-modification method is that the proteins are subjected to highly acidic conditions (pH < 1), which may strongly reduce the solubility of proteins and, as a consequence affect the reactivity of the citrulline residues. To retain the citrullinated proteins in a more exposed state and to circumvent the use of AMC antibodies, we explored a strategy in which the proteins are first separated by gel electrophoresis and blotted on a membrane and then modified by incubation with an azide-containing PG molecule (4-azido-PG) at low pH. Subsequently, the reaction products can be visualized with alkyne-containing probes that are readily available with different reporter molecules. We here show that this ‘on-blot’ procedure is a robust and sensitive alternative for AMC antibodies to detect citrulline residues in purified proteins and in complex protein samples like cell lysates. 

## 2. Results and Discussion

**Figure 1:**
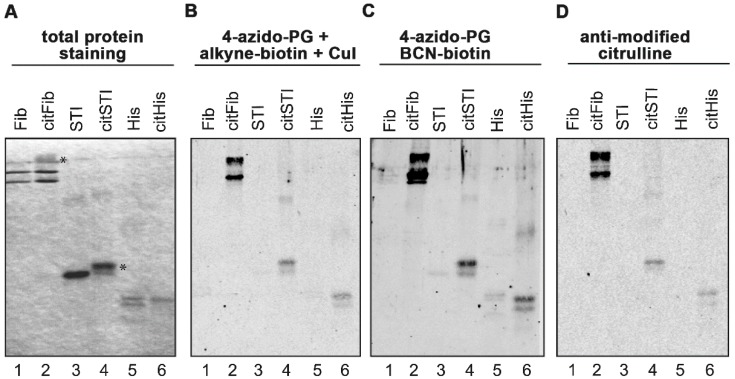
PG-based detection of citrullinated proteins on membranes. Fibrinogen (Fib), soybean trypsin inhibitor (STI) and histones (His) were citrullinated *in vitro* by PAD in the presence of calcium. Proteins were separated by SDS-PAGE and stained with Coomassie Brilliant Blue (**A**). Some citrullinated proteins showed reduced migration in the gel, likely due to the charge difference of arginine and citrulline (panel A, indicated with an asterisk). Proteins were transferred to nitrocellulose membranes and the resulting blots were incubated for 3 h with 1 mM 4-azido-PG and subsequently for 1 h with 10 μM alkyne-biotin (**B**) or 10 μM BCN-biotin (**C**). Biotinylated reaction products were visualized with Neutravidin DyLight 800. For the AMC procedure the membrane was incubated with DAMO and antipyrine under highly acidic conditions and chemically modified proteins were detected by anti-modified citrulline antibodies (**D**).

**Scheme 1:**
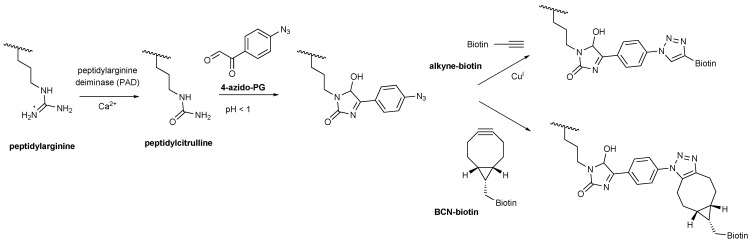
4-Azido-PG based detection of peptidylcitrulline. Peptidylarginine is converted to peptidylcitrulline by PAD enzymes. Under highly acidic conditions peptidylcitrulline reacts specifically with 4-azido-PG. Alkyne-biotin was used for the copper-catalyzed (CuAAC) and BCN-biotin for the strain-promoted (SPAAC) azide-alkyne cycloaddition to visualize the peptidylcitrulline-PG reaction products.

To explore the suitability of the ‘on-blot’ citrulline-modification procedure human fibrinogen (Fib), soybean trypsin inhibitor (STI) and histones from calf thymus (His) were citrullinated *in vitro* by incubation with rabbit muscle PAD in the presence of calcium. Both the citrullinated and untreated proteins were separated by SDS-PAGE and either directly stained by Coomassie Brilliant Blue (Figure 1A) or transferred to nitrocellulose membranes (Figure 1B–D). Optimized conditions were used for the modification of the citrullinated proteins (optimization of the conditions is shown in the supplementary materials Figures S1–S3). Membranes were incubated for 3 h with 1 mM 4-azido-PG in a solution containing 25% trifluoroacetic acid (TFA) to allow the glyoxal moiety to react with the ureido group of the citrulline residues (Scheme 1). 

**Figure 2:**
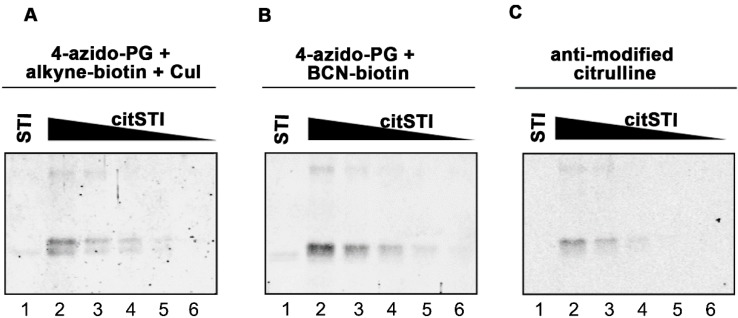
Sensitivity of PG-based detection of citrullinated proteins. A two-fold dilution series of citrullinated STI (200–12.5 ng) was prepared and proteins were separated by SDS-PAGE and transferred to nitrocellulose membranes. As a control 200 ng non-citrullinated STI was analyzed in parallel (lane 1). Blots were incubated for 3 h with 1 mM 4-azido-PG and subsequently for 1 h with 10 µM alkyne-biotin (**A**) or 10 µM BCN-biotin (**B**). Biotinylated reaction products were visualized with Neutravidin DyLight 800. For the AMC procedure the blot was incubated with DAMO and antipyrine under highly acidic conditions and chemically modified proteins were detected by anti-modified citrulline antibodies (**C**).

Before labeling with 4-azido-PG, the sulfhydryl groups of the proteins on the membranes were blocked by incubation with 10 mM iodoacetamide to avoid undesired thiol-yne reactions with the cysteines [5,6]. The reaction products were labeled with 10 μM of an alkyne-containing biotin probe by either copper(I)-dependent azide-alkyne cycloaddition (CuAAC) or strain-promoted azide-alkyne cycloaddition (SPAAC) with a bicyclo[6.1.0]nonyne biotin (BCN-biotin, Scheme 1). The nitrocellulose membranes were subsequently blocked with bovine serum albumin (BSA) and incubated with IRdye-labeled neutravidin. To determine the suitability of these methods, the citrullinated proteins were also stained by the AMC method (Figure 1D). The signals obtained with the 4-azido-PG-based methods appeared to be similar to the signals obtained with the AMC method. To compare their sensitivities, membranes containing decreasing amounts of citrullinated STI were subjected to the respective procedures (Figure 2). The alkyne-biotin method showed similar sensitivity as the AMC method, while the BCN-biotin method was slightly more sensitive. With all three approaches 25 ng of citrullinated STI (~1 pmol) could be detected.

**Figure 3:**
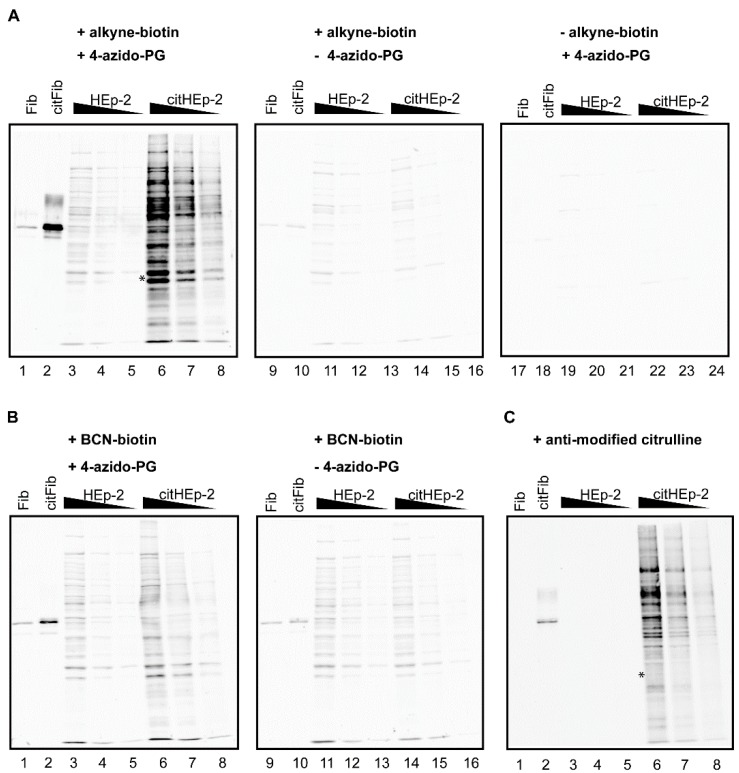
PG-based detection of citrullinated proteins in a cell extract. A HEp-2 cell extract was incubated with PAD and calcium to citrullinate proteins present in the extract (citHEp-2). A mock-modified extract to which no PAD enzyme was added was analyzed in parallel (HEp-2). Non-citrullinated (Fib) and citrullinated fibrinogen (citFib) were used as controls (400 ng). Total protein extracts (5 μg, 2.5 μg, and 1.25 μg) were separated by SDS-PAGE and transferred to nitrocellulose membranes. Blots were incubated for 3 h with 1 mM 4-azido-PG (**A**: lanes 1–8; 17–24 and **B**: lanes 1–8) or no 4-azido-PG (A and B: lanes 9–16) and subsequently for 1 h with 10 μM alkyne-biotin (A: lanes 1–16) or 10 μM BCN-biotin (B: lanes 1–16). Biotinylated reaction products were visualized with Neutravidin DyLight 800. For the AMC procedure the membrane was incubated with DAMO and antipyrine under highly acidic conditions and chemically modified proteins were detected by anti-modified citrulline antibodies (**C**).

We next investigated whether the 4-azido-PG-based methods are also suitable for the detection of citrullinated proteins in complex biological samples. For this, blots containing different amounts of non-citrullinated or *in vitro* citrullinated HEp-2 cell extracts were used. The blots were incubated with 4-azido-PG followed by either alkyne-biotin (Figure 3A) or BCN-biotin (Figure 3B). As controls, 4-azido-PG or alkyne-biotin were omitted. Furthermore, a blot containing the same material was subjected to the AMC procedure (Figure 3C).

The citrullinated proteins present in the cell extract were readily detected by the alkyne-biotin and the AMC method (Figure 3, citHEp-2). However, the alkyne-biotin based method also showed detectable signals for non-citrullinated proteins, which means that with this method proper control analyses should always be performed in parallel. The signals for the citrullinated proteins obtained by the BCN-biotin method were only slightly above the signals obtained for the non-citrullinated proteins, which makes this method less suited for complex biological samples.

A comparison of the results indicated that the alkyne-biotin method allowed the detection of several citrullinated proteins, which were not or less well detectable with the AMC method (Figure 3, compare lanes 6 in panel A and C). This is most obvious in the region indicated with the asterisk: the two bands present in lane 6 of panel A are not visible in lane 6 of panel C. This suggests that not all peptidylcitrulline residues are recognized equally well by these two methods. The antibodies used in the AMC method were obtained by immunizing rabbits with DAMO/antipyrine-modified histones [3]. The possibility exists that the interaction between AMC and the DAMO/antipyrine-modified citrulline is influenced by the surrounding amino acids, which means that AMC might have a preference for certain citrulline residues. A similar amino acid context preference for the reaction of phenylglyoxal with the ureido group in the peptidylcitrulline side chain is not very likely and the very strong interaction between neutravidin and biotin is not expected to be affected by the surrounding amino acids of the target proteins. Further research will be required to establish whether the alkyne-biotin method gives a better reflection of the citrullinated proteins present in a cell extract than the AMC method.

As already mentioned above, another post-translational modification, carbamylation, the conversion of peptidyllysine into peptidylhomocitrulline, results in the generation of a chemical moiety that is very similar to that of citrulline. Carbamylation occurs when proteins are exposed to cyanate, which may occur under certain (patho)physiological conditions [7]. Due to the chemical similarity of the citrulline and homocitrulline side chains, phenylglyoxal will, like DAMO used in the AMC method, react with both of these amino acids and therefore these methods will not discriminate between citrullination and carbamylation.

## 3. Experimental Section

### 3.1. Alkyne-Biotin

Biotin-NHS (25 mg, 73 µmol) was dissolved in DMSO (659 μL). Propargylamine (65 µmol, 3.6 mg) was added and the mixture was rotated overnight at room temperature [8]. 1 M Tris-HCl, pH 7.6 (300 μL) was added to block unreacted biotin-NHS and the mixture was rotated for 1 h at room temperature. The synthesis of propargyl-biotin was confirmed by LC-MS analysis. [M+H]^+^ calculated for C_13_H_20_N_3_O_2_S: 282.1; found: 282.4. The propargyl-biotin product was used without further purification.

### 3.2. In Vitro Citrullination

Human fibrinogen (25 μg, Sigma-Aldrich, Saint Louis, MO, USA), soybean trypsin inhibitor (25 μg, Sigma-Aldrich), and histones from calf thymus (25 μg, Calbiochem, Darmstadt, Germany) were citrullinated *in vitro* by incubation with rabbit skeletal muscle PAD (75 mU; Sigma-Aldrich; EC 3.5.3.15) in deimination buffer (40 mM Tris-HCl, pH 7.5, 5 mM CaCl_2_ and 1 mM dithiothreitol) at 37 °C for 3 h. The reaction was stopped by the addition of EGTA, pH 8.0, to a final concentration of 50 mM. Unless indicated otherwise, 400 ng of protein was loaded per lane on polyacrylamide gel. For the *in vitro* citrullination of proteins present in HEp-2 cells, 2.5 × 10^6^ cells were resuspended in 200 μL lysis buffer (20 mM Tris-HCl, pH 7.6, 100 mM NaCl, 10 mM β-mercaptoethanol, 10% glycerol, Complete EDTA-free protease inhibitor cocktail mix (Roche, Woerden, Netherlands) and homogenized by sonication, after which cellular debris was removed by centrifugation. CaCl_2_ (final concentration 10 mM), dithioerythritol (final concentration 5 mM) and rabbit muscle PAD (250 mU) were added to the lysate (500 μg total protein) and the mixture was incubated for 3 h at 37 °C. The reaction was stopped by the addition of EGTA, pH 8.0 (final concentration 5 mM). A parallel incubation of a lysate to which no PAD enzyme was added served as a negative control.

### 3.3. Detection of Citrullinated Proteins by 4-Azido-PG and Azide-Alkyne Cycloaddition on Membranes

Proteins were separated by SDS-PAGE (10% or 15% polyacrylamide) and transferred to Hybond C-extra nitrocellulose membranes (Amersham Biosciences, Uppsala, Sweden) by electroblotting. Cysteines were blocked by incubating the blots with freshly prepared iodoacetamide (10 mM in PBS) for 1 h in the dark at room temperature. After washing with PBS/0.05% Tween-20 (PBS-T), blots were incubated with 1 mM 4-azido-PG (Apollo Scientific, Cheshire, UK) in TFA (25% in PBS-T) for 3 h at room temperature. Blots were washed with PBS-T (3 × 15 min) and incubated with 10 μM alkyne-biotin supplemented with CuSO_4_ (10 mM), sodium ascorbate (10 mM) and tris(1-((O-ethyl)carboxymethyl)-(1,2,3-triazole-4-yl))methylamine [9] (triazole ligand; 10 mM) in PBS-T or 10 μM BCN-biotin [10] in PBS-T for 1 h at room temperature. Subsequently, blots were washed again with PBS-T (3 × 15 min) and blocked for 1 h at room temperature with BSA (5% in PBS-T). Biotinylated reaction products were visualized by incubation with Neutravidin DyLight 800 (1:15,000, Pierce, Paisley, UK) in PBS-T containing 5% BSA and scanned using an Odyssey infrared scanner (LI-COR, Lincoln, NE, USA).

### 3.4. Detection of Citrullinated Proteins by the Anti-Modified Citrulline Antibodies

Proteins were separated by SDS-PAGE (10% or 15% polyacrylamide) and transferred to Hybond C-extra nitrocellulose membranes (Amersham Biosciences) by electroblotting. Blots were blocked for 15 min with ovalbumin (0.1% in PBS), rinsed with demineralized water and subsequently incubated with paraformaldehyde (4% in PBS) for 15 min. Blots were rinsed again with demineralized water and chemically modified by overnight incubation at 37 °C with a solution containing DAMO (0.25% (*w*/*v*)), antipyrine (0.125% (*w*/*v*)), FeCl_3_ (0.0125% (*w*/*v*)), acetic acid (0.25 M), H_2_SO_4_ (2.3 M) and H_3_PO_4_ (1.5 M). After thoroughly washing the blots with PBS, they were incubated with anti-modified citrulline antibodies (1:1,000, Millipore, Darmstadt, Germany) in PBS-T containing 5% non-fat dried milk for 3 h at room temperature. Finally, the blots were washed with PBS-T and bound antibodies were visualized with IRDye 800CW goat anti-rabbit IgG (LI-COR) for 1 h at room temperature and scanned using a LI-COR Odyssey infrared scanner.

## 4. Conclusions

We conclude that the method based on 4-azido-PG in combination with alkyne-biotin cycloaddition, and possible other alkyne-conjugated probes, provides a good antibody-independent alternative for the detection of citrullinated proteins on membranes. The labeling reaction is performed after the proteins are transferred to the membrane, to allow optimal exposure of the proteins to the reagents under the highly acidic conditions and to prevent aberrant effects due to precipitation. Furthermore, the alkyne-biotin method may give a better reflection of the citrullinated proteins present in cell extracts than the AMC method, because the method is antibody independent. The versatility of this two-step cycloaddition-chemistry makes this a very attractive approach. The probe can be easily adapted to alternative visualization procedures, such as those that are based on other fluorescent or chemiluminescent compounds.

## Supplementary Materials

Supplementary materials can be accessed at: http://www.mdpi.com/1420-3049/20/04/6592/s1.
