# Supplementary material for: Phenylglyoxal-Based Visualization of Citrullinated Proteins on Western Blots
Source: Molecules. 2015 Apr 14;20(4):6592–600. doi: 10.3390/molecules20046592 (PMC6272700; doi:10.3390/molecules20046592)
Supplement: Supplementary file 1 [file molecules-20-06592-s001.pdf]

Supplementary Materials

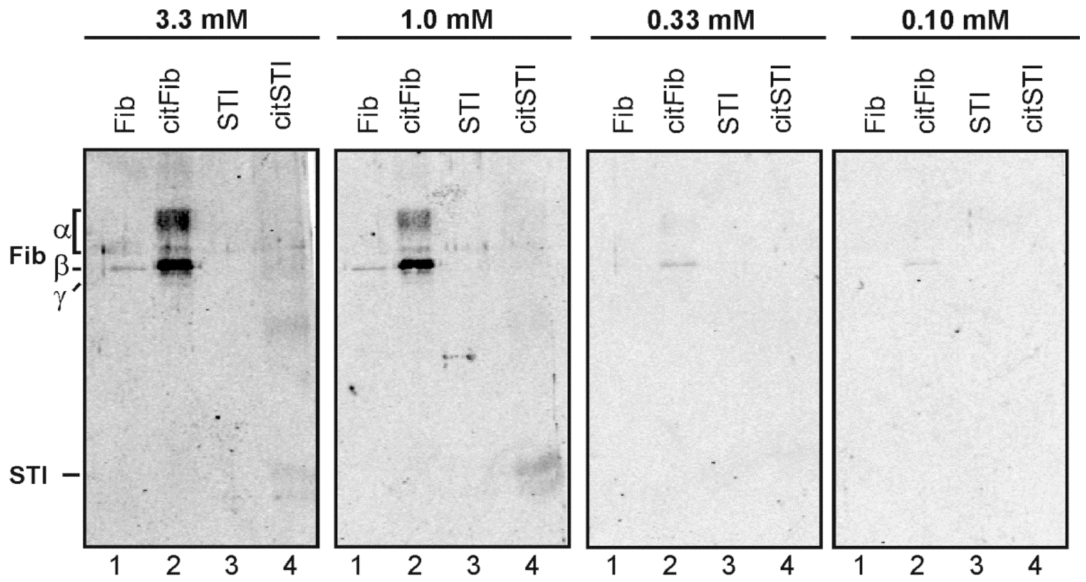

Figure S1. Optimization of the 4-azido-PG concentration for the detection of citrullinated proteins on western blots. Fibrinogen (Fib) and soybean trypsin inhibitor (STI) were citrullinated in vitro by PAD in the presence of calcium. Proteins were separated by SDS-PAGE and transferred to nitrocellulose membranes. Blots were incubated for 3 h with 3.3 mM, 1.0 mM, 0.33 mM or 0.10 mM 4-azido-PG and subsequently with 10 µM alkyne-biotin in the presence of CuI. Biotinylated reaction products were visualized with Neutravidin DyLight 800. The positions of the (citrullinated) fibrinogen α, β and γ chains and of STI are indicated on the left. The incubation with 1 mM 4-azido-PG was selected as the optimal condition.

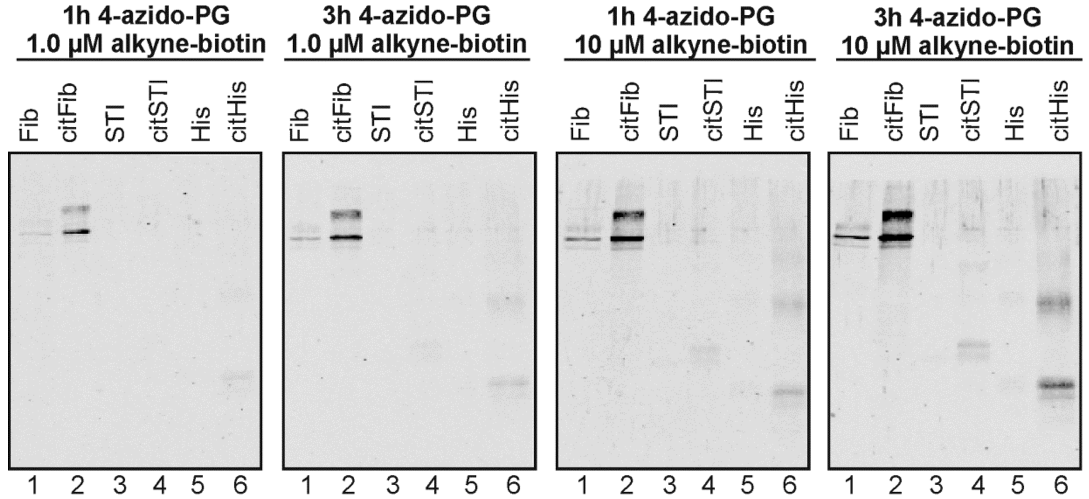

Figure S2. Optimization of the alkyne-biotin concentration and 4-azido-PG incubation time. Fibrinogen (Fib), soybean trypsin inhibitor (STI) and histones (His) were citrullinated in vitro by PAD in the presence of calcium. Proteins were separated by SDS-PAGE and transferred to nitrocellulose membranes. Blots were incubated for 1 or 3 h with 1 mM 4-azido-PG and subsequently with 1.0 µM or 10 µM alkyne-biotin in the presence of Cu^{I}. Biotinylated reaction products were visualized with Neutravidin DyLight 800. The 3 h incubation with 10 µM alkyn-biotin was selected as the optimal condition.

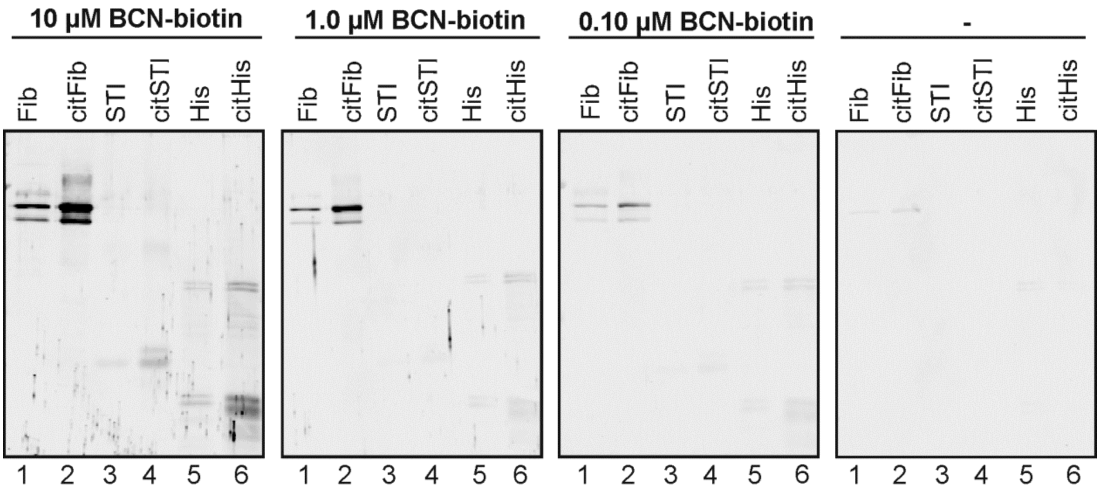

Figure S3. Optimization of the BCN-biotin concentration. Fibrinogen (Fib), soybean trypsin inhibitor (STI) and histones (His) were citrullinated in vitro by PAD in the presence of calcium. Proteins were separated by SDS-PAGE and transferred to nitrocellulose membranes. Blots were incubated for 3 h with 1 mM 4-azido-PG and subsequently with 10 μM, 1.0 μM, 0.10 μM or no BCN-biotin. Biotinylated reaction products were visualized with Neutravidin DyLight 800. The incubation with 10 μM BCN-biotin was selected as the optimal condition.
